# Development of an adjustment ability scale for outpatients with cancer: Verification of its reliability and validity

**DOI:** 10.1002/nop2.145

**Published:** 2018-04-15

**Authors:** Keiko Hirokawa, Shizue Suzuki

**Affiliations:** ^1^ Kawasaki University of Medical Welfare Kurashiki Okayama Japan; ^2^ Kobe City College of Nursing Kobe Hyogo Japan

**Keywords:** ability, adjustment, nurses, nursing, outpatient, reliability and validity

## Abstract

**Aims:**

To develop an adjustment ability scale for outpatients with cancer and to investigate its reliability and validity.

**Design:**

Quantitative study.

**Methods:**

A proposed adjustment ability scale was prepared based on scale development guidelines. Its reliability and validity were statistically analysed using data obtained from 369 patients.

**Results:**

Six factors were extracted from the factor analysis. Cronbach's α coefficient was 0.95 and the test–retest reliability coefficient was 0.83. A correlation coefficient of .48 was determined between the adjustment ability scale and the Mental Adjustment to Cancer scale (Japanese version), which assesses psychological adaptations via the cognitive‐behavioural responses of patients with cancer. The correlation coefficient between the scale and the Functional Assessment of Cancer Therapy‐General (Japanese version 4), which measures quality of life among patients with cancer, was 0.15.

## 1 INTRODUCTION

The number of outpatient cancer treatments has increased due to shorter hospitalization stays and increases in outpatient chemotherapy and radiotherapy rates. Patients with cancer also need to receive periodic examinations because cancer is characterized by metastasis and recurrence. In Japan, 57.0% of patients with cancer receive treatment and periodic examinations as outpatients (Statistics and Information Department, Minister's Secretariat, Ministry of Health, Labour and Welfare, 2016). After discharge, cancer patients have various concerns including physical condition, feelings about cancer, lifestyle, information, stigma and economy (Dodd, Dibble, & Thomas, 1992; Oberst & James, 1985). Most outpatients with cancer have symptoms associated with surgery, metastasis and recurrence (Kondo, Shimizu, Watanabe, Fukuda, & Oishi, 2004; Naka, Oishi, & Onishi, 2007) and their daily life is markedly affected by adverse events (Lai, Ching, & Wong, 2017). Moreover, the relationship between outpatients with cancer and other people changes because their social roles have been changed or reduced as a result of their periodic hospital visits and their treatments (Muraki & Onishi, 2006; Narui et al., 2004). In other words, outpatients with cancer have to adjust to various changes in their life with cancer onset and treatment.

## 2 BACKGROUND

Lazarus and Folkman (1991) developed a psychological stress model to show that coping actions can be determined by people's cognitive assessment of stressful events. This model has been widely used in nursing practice. In particular, outpatients have to cope with stressful situations by themselves and how they cope with these situations has attracted attention. Some studies have focused on what cancer patients believe is difficult in specific outpatient treatment situations and how to cope with these difficulties (Takayama, 2016; Tanaka & Tanaka, 2012), while others have focused on how to cope with side effects associated with new treatments (McSorley et al., 2013; Speck et al., 2013; Takei, Seyama, Ishida, & Kanda, 2011). The scales currently used to evaluate coping actions include the Ways of Coping Questionnaire, which was developed in the theoretical context of the psychological stress model and the COPE and Brief COPE, which were developed based on the psychological stress model and the model of self‐regulation. These scales have been used in studies of cancer patients (Manuel et al., 2007; Wonghongkul, Moore, Musil, Schneider, & Deimling, 2000) and mixed methods research studies (McSorley et al., 2013).

I hypothesized that outpatients with cancer not only cope with difficulties and issues that they recognize, but also continue to try to control their physical conditions and arrange their life in general. Therefore, I investigated what outpatients with cancer cope with inductively and qualitatively (Hirokawa, 2016). That investigation revealed that these patients cope in a way that can be explained by the psychological stress model and they want to learn about other coping strategies and better ways to cope with their situations. These patients also made an effort to establish the criteria necessary to find a specific way to cope. This result suggested that outpatients with cancer prepare or make necessary arrangements for an appropriate coping response in addition to coping with difficulties and issues they recognize, in a way that can be explained by the psychological stress model.

Most studies have focused on the ability of outpatients to cope with difficulties, since it is harder for them to consult with medical professionals promptly because they live at home instead of staying at the hospital, they have to rearrange their life and each individual's living situation is different (Asano & Sato, 2002; Sato & Sato, 2010). Conversely, their ability to control physical conditions and arrange their life in general after cancer onset has not been investigated. The establishment of a scale to evaluate outpatients’ life adjustment ability after cancer onset may help us understand their adjustment ability and may lead to discussions and evaluations of nursing strategies in outpatient cancer nursing practices.

In this study, outpatients with cancer are defined as “those who visit an outpatient department regularly for follow‐up or continuous treatment.” Adjustment ability is defined as the “ability of the patient him/herself to adjust to a situation requiring cognitive‐behavioural efforts to keep mental and physical balances and live by his/her own values after cancer onset. This ability will gradually improve through continued use.”

## 3 METHODS

### 3.1 Preparation of proposed adjustment ability scale (DeVellis, 2003)

#### 3.1.1 Extraction of adjustment ability components

Thirteen outpatients with cancer were interviewed to extract adjustment ability components: “Ability to think”; “Ability to act”; “Ability to evaluate”; “Ability to recognize and understand coping mechanisms”; “Ability to change objectives” and “Ability to expand the ways you adjust” (Hirokawa, 2016).

#### 3.1.2 Preparation of item pool

An item pool was prepared based on a code for each adjustment ability component. Also, other items were added to prepare a total of 83 item pools based on experience recordings (Hata, 1996; Inako, 1996; Yoshikawa, 1999; Ohashi, 2005; Ohashi, 2008), previous studies (Akaishi, Fuse, & Kanda, 2004; Horii, Kobayashi, & Suzuki, 2009; Kosaka & Majima, 2011; Miyazaki, Hata, Iwashita, Hidaka, & Morishita, 2008; Mori & Akimoto, 2005; Nakamura & Kamizato, 2010; Nakao, 2005; Nakazawa, Kanda, Kyota, & Honda, 2014; Okamoto & Sato, 2008; Tanaka & Tanaka, 2012) and the experiences of researchers.

#### 3.1.3 Assessment of validity and usability

The content‐related validity of the 83 question items was assessed according to the method developed by Imle and Atwood (1988) with the cooperation of 11 medical personnel including nurses who specialize in cancer care and university professors who have a master's degree and are involved in cancer care nurses. Among responses obtained from four phases, the percentage of those who responded with “very much” and “almost” was calculated as the rate of concordance (%) and the rate equal to or higher than 80% was the numerical criterion for analysis. The number of question items was changed from 83 to 67 based on the analysis.

Then, the face validity of 67 question items was assessed with the cooperation of 10 cancer patients using my personal network. As a result of the assessment, no items needed to be deleted or revised. Patients were requested to provide feedback about questions that were difficult to answer or hard to understand. Based on this feedback, two question items were reworded.

#### 3.1.4 Pilot study

A self‐completed questionnaire that included 67 question items from the proposed adjustment ability scale was sent to 113 people with cancer; 59 patients responded (collection rate 52.2%). Among those 59 responses, 58 were considered valid (valid response rate 51.3%). Three question items with an item‐total (IT) correlation coefficient lower than .20 were revised: 1) “If you have any annoying symptom or problem, you will consult a doctor or nurse,” was changed to “If you have any symptom or problem that you cannot cope with, you will consult a doctor or nurse,” to emphasize that the problem is difficult for the patient to cope with; 2) “If you are depressed, you can control yourself to some extent to not be depressed anymore,” was revised to “If you are depressed, you can take actions to stop becoming depressed further,” because taking actions to stop becoming even more depressed is considered to be more realistic; and 3) “If it is difficult for you to decide something, you can consult with someone,” was changed to “If it is difficult for you to decide something by yourself, you can consult with someone,” to emphasize that it is difficult for the patient to cope by him/herself. Cronbach's α coefficient across the 67 question items was 0.920 and the test–retest correlation coefficient across the scale was .557, which demonstrates the scale's reliability. Based on the results described above, a proposed adjustment ability scale consisting of 67 question items was prepared.

### 3.2 Items to be investigated

#### 3.2.1 Patient characteristics

The patient characteristic categories included sex, age, cancer location and duration from the first cancer diagnosis. Also, 12 items were investigated to confirm whether a patient had any difficulty in his/her life including diet, bowel movements and urination.

#### 3.2.2 Proposed adjustment ability scale

The proposed adjustment ability scale with 67 question items was used. Patients looked back on their experiences from their first cancer diagnosis to the present situation to grade themselves based on a five‐point scale: “Very much true = 4”; “quite a bit true = 3”; “Somewhat true = 2”; “A little bit true = 1” and “Not true at all = 0”. Scores for each item were added to calculate a total adjustment ability score.

#### 3.2.3 Scales used to investigate validity

##### Mental adjustment to cancer scale (Japanese version)

The mental adjustment to cancer (MAC) scale was developed by Watson et al. (1988) as a tool to study the coping abilities of patients receiving cancer treatment. It has been used in studies of patients with breast cancer to show how they cope with the disease (Carlsson, Arman, Backman, & Hamrin, 2001; Watson, Haviland, Greer, Davidson, & Bliss, 1999). This scale has five subscales with a total of 40 items to evaluate the psychological adjustment ability of patients with cancer through cognitive‐behavioural coping skills: fighting spirit (16 items), anxious preoccupation (9 items), fatalism (8 items), helplessness/hopelessness (6 items) and avoidance (1 item). It is a four‐point scale ranging from “Definitely not” to “Absolutely true”. Akechi et al. (1997) verified the reliability and validity of the Japanese version.

Researchers believe that patients with cancer who have a higher adjustment ability can balance mental and physical well‐being by effectively controlling physical conditions, social communications, economic situations and mental/psychological conditions in their daily lives. Therefore, I hypothesized that there was a positive correlation between the adjustment ability scale score, the MAC scale and the fighting spirit score, which was the most effective among the subscales and correlated with a patient's recognition of being well in control of his/her cancer and mental/physical conditions. I also hypothesized that there was a negative correlation between the adjustment ability score and the helplessness/hopelessness score, which was considered to be the most adverse coping action.

##### Functional assessment of cancer therapy‐general (Japanese version 4)

The functional assessment of cancer therapy‐general (FACT‐G) is a questionnaire developed by Cella et al. (1993) to measure the quality of life (QOL) of patients with cancer. Its reliability and validity have already been demonstrated. It is composed of four subscales with a total of 27 items: physical (7 items), social/familial (7 items), psychological (6 items) and functional (7 items) aspects. Patients rate on a scale of 1‐5 (from “Not true at all” to “Very much true”). The Japanese version was developed by Fumimoto et al. (2001) and its reliability and validity have been demonstrated Shimotsuma and Eguchi (2001).

It is expected that patients can balance mental and physical well‐being and live by their own values with a better QOL if they have a higher adjustment ability. However, the adjustment ability graded on this scale is based on a patient's own ability and I believe the association with QOL evaluated by the FACT‐G was weak.

### 3.3 Institutions and participants

I asked 19 medical institutions located in western Japan to participate in the study. The inclusion criteria for this study were cancer patients 20–70 years old who were informed that they had cancer and those who visited an outpatient department for follow‐up or continuous treatment after more than 6 months from discharge after the initial treatment. These criteria were chosen for the development of a widely applicable scale without specifying the type of cancer (first or recurrent) and treatment regimen.

### 3.4 Data collection method

After obtaining study participation agreement from responsible persons in the medical institutions, I asked nurses in outpatient departments for their cooperation in participant selection. I provided an explanation about the purpose and method of the study to the participants and gave them a questionnaire form for the test and then another for the retest with a reply envelope. Participants were asked to return the completed retest form 2 weeks after the first response. The data were collected between September 2013 ‐ August 2015.

### 3.5 Analytical methods

#### 3.5.1 Scale development

Forms with a blank answer field in less than 10% of the total number of question items were considered valid; a mean score was inserted in the blank fields (Polit & Beck, 2010). The mean ± *SD* score was calculated for each question item to examine ceiling and floor effects. Adjustment ability scale scores were classified as higher score (top 25% of the total score) or lower score (bottom 25% of the total score) groups to conduct a *t* test for the mean score for each question item (a total of 67 items). Question items were selected by using a reference value of .30 for the IT correlation coefficient and .07 for Spearman's rank correlation coefficient between items. The number of factors was determined by a scree plot and a cumulative contribution ratio greater than 50%. Because I assumed that adjustment ability is expressed through a combination of the abilities represented by the subscales, I conducted a factor analysis by promax rotation and the method of least squares, assuming there was a correlation between factors. I excluded items with a factor loading value lower than 0.35 and those with a higher load across two factors, analysed repeatedly to extract factors and named question items based on item details. I calculated the mean score across the scale, the range, median and total scores of the scale, the subscale score and Spearman's rank correlation coefficient between subscales.

#### 3.5.2 Verification of reliability

Cronbach's α confidence coefficient was calculated across the adjustment ability scale and for each subscale and the internal consistency was validated. Stability was examined based on Spearman's rank correlation coefficient, the correlation coefficient across the adjustment ability scale and for each subscale and the intraclass correlation coefficient in test and retest items.

#### 3.5.3 Verification of validity

Spearman's correlation coefficient was calculated for the adjustment ability scale, MAC scale, fighting spirit, helplessness/hopelessness and FACT‐G scores. A confirmatory factor analysis was conducted to calculate the model congruence index (goodness‐of‐fit index [GFI], adjusted goodness‐of‐fit index [AGFI], comparative fit index [CFI] and root mean square error of approximation [RMSEA]). The analysis was conducted using SPSS22.0J (IBM SPSS Amos Authorized User version 21).

### 3.6 Ethical considerations

Before the study began, it was approved by author's affiliation Ethics Committee as well as the ethics committees of participating institutions. I provided study participants with oral and written explanations that their participation in this study was entirely voluntary and not associated with any medical institutions. Anonymous questionnaires were collected by mail and final study participation consent was considered to be obtained when a questionnaire was returned. Completed questionnaires were handled with care to protect personally identifiable information.

## 4 RESULTS

### 4.1 Participant selection

A total of 750 questionnaire forms were sent to 15 institutions: 11 linked regional core centres for the treatment of cancer; one prefectural‐designated hospital for the treatment of cancer; two general hospitals and one clinic. A total of 409 participants responded (collection rate 54.5%). Among these responses, 369 were considered to be valid (valid response rate 49.2%). Questionnaire forms for the retest were sent to 750 participants; 189 participants responded (collection rate 25.2%). Among those responses, 170 were considered to be valid (valid response rate 22.7%).

### 4.2 Participant characteristics (Table 1)

**Table 1:** Participant characteristics

Sex		
Female	262	71.0
Male	107	29.0
Age		
20s	1	0.3
30s	19	5.1
40s	68	18.4
50s	105	28.5
60s	176	47.4
Mean	56.61 ± 9.33	
Range	25–69	
Location of cancer (multiple answers allowed) (one no response)		
Respiratory organ	70	19.0
Gastrointestinal tract	88	23.8
Liver, gallbladder, bile	32	8.7
duct and pancreas	11	3.0
Urinary organ	7	1.9
Prostate	186	50.4
Breast	19	5.1
Female genital organs	2	0.5
Blood	38	10.3
Others	301	81.6
Only 1 ≥ 2	67	18.2
Duration from the first diagnosis		
≥6 months–<1 year	55	14.9
≥1–<2 years	68	18.4
≥2–<3 years	50	13.6
≥3–<4 years	48	13.0
≥4–<5 years	28	7.6
≥5–<8 years	63	17.0
≥8–<10 years	20	5.5
≥10–<20 years	29	7.9
≥20 years	8	2.2
Mean	4 years and 6 months ± 4 years and 11 months	
Range	6 months–43 years and 10 months	
Difficulties in physical functioning and daily life due to cancer and treatment (multiple answers allowed)		
With difficulties	328	88.9
Diet	228	61.8
Bowel movement and urination	180	48.8
Physical movement	236	64.0
Retaining posture	133	36.0
Sleep	203	55.0
Bathing	130	35.2
Putting on, taking off and	121	32.8
Selection of clothes	92	24.9
Communication	120	32.5
Sex	143	38.8
Communication with others	186	50.4
Money	131	35.5
Hobbies	40	10.8
Without difficulties	31	8.4
No response (total)		

The participants of this study included 262 women (71.0%) and 107 men (29.0%) with an age range of 25–69 years (mean age 56.6 years). Cancer location included breast in 186 participants (50.4%) and the gastrointestinal tract in 88 participants (23.8%; oesophagus, stomach and bowel in descending order of frequency). The duration from the first diagnosis ranged from 6 months to 43 years and 10 months (mean duration 4.5 years). With respect to difficulties in physical functioning and daily life due to cancer and treatment, 328 participants (88.9%) responded that they had difficulties, while 40 participants (10.8%) said that they had no difficulties.

### 4.3 Scale development

None of the 67 question items had a suspected ceiling effect or floor effect. The mean score for the higher score group was significantly higher than the lower score group for all question items (*p* < .001). One question item with an IT correlation coefficient lower than .30 (reference value) was deleted (*p* = .257). Two items had a Spearman's rank correlation coefficient of .70 or higher between items and one of them was deleted because it showed a correlation coefficient of .60 or higher with other items.

An exploratory factor analysis was conducted for 65 question items. In this analysis, six factors were used because Factor VI had the largest eigenvalue difference in the scree plot and the cumulative contribution ratio from Factors I to VI was 52.9%. After repeated analyses excluding items based on factor loadings, 18 question items were excluded, and six factors and 47 items were selected (Table 2). The factors were named the following: Factor I = “Ability to tell someone”; Factor II = “Ability to seek a better way to cope”; Factor III = “Ability to increase certainty”; Factor IV = “Ability to recognize and understand coping mechanisms”; Factor V = “Ability to change objectives” and Factor VI = “Ability to control physical function”.

**Table 2:** Results of exploratory factor analysis

Name of factor: Question item	Factor loading					
	I	II	III	IV	V	VI
Factor I: Ability to tell someone						
You tell people around you your feelings	0.881	−0.015	−0.133	0.018	−0.025	−0.081
You tell people around you that you are in bad physical condition to avoid a misunderstanding	0.664	−0.114	0.035	0.124	0.005	−0.103
You ask people around you to do something that you think you cannot do physically or it is physically strenuous work	0.650	−0.008	−0.123	−0.091	−0.001	0.220
You consult with people around you or ask them to help you change your traditional ways when necessary	0.647	0.090	0.101	−0.114	0.031	0.024
You tell your colleagues or family about your planned hospital visit a day early	0.640	−0.131	0.195	0.082	−0.104	−0.002
If you are in bad physical condition, you tell people around you about it	0.633	0.031	−0.214	−0.076	0.013	0.146
You ask your colleagues or family to help you deal with your hospital visit and work or domestic duties	0.631	−0.111	0.212	0.042	−0.100	0.027
You consult with a doctor or nurse if you cannot cope with your physical condition or if you have any questions	0.588	0.011	0.021	0.040	−0.001	0.099
You have someone to confide in if you have difficulties coping by yourself	0.489	0.027	−0.035	0.033	0.138	−0.081
You talk about your physical condition in detail to your attending doctor	0.467	0.144	−0.064	0.015	−0.085	0.090
You express your appreciation to those who support you	0.427	0.027	0.173	−0.080	0.251	−0.101
Factor II: Ability to seek a better way to cope						
You think about whether things that you are doing for your health are really good or not	0.002	0.856	−0.052	0.054	−0.047	−0.144
You think about the physical burden associated with a new way of coping	0.062	0.763	−0.037	0.036	0.004	−0.006
You think about better ways to cope, even if you can almost cope	0.153	0.761	−0.088	−0.082	−0.006	−0.087
You think about how to continue doing what you believe is good for your health	−0.091	0.761	0.011	−0.024	−0.002	0.018
You think about how to improve your strength gradually without physical burden	−0.241	0.713	0.027	−0.063	0.030	0.171
You keep an eye out for physical changes	−0.109	0.636	0.083	−0.023	−0.053	0.058
You try to do what you think is necessary for a stable physical condition and life	0.048	0.586	−0.116	−0.036	0.063	0.203
If your physical condition is different than usual, you think about possible causes	−0.037	0.514	0.205	−0.031	−0.099	0.026
You think about whether you get enough rest	0.133	0.491	0.090	0.092	0.006	−0.054
You think about reasons why you could or could not cope with something	0.112	0.490	0.205	0.133	−0.027	−0.059
You continue to have the same life rhythm if you keep in good physical condition	0.091	0.436	0.042	0.014	−0.043	0.184
You think about whether you should continue to change the way you manage yourself according to your physical condition	0.185	0.415	0.143	0.095	−0.058	0.115
Factor III: Ability to increase certainty						
You think about when and where to rest so that people around you do not worry about you if you are in poor physical condition	−0.158	−0.002	0.845	−0.023	0.061	0.003
You think about how to prepare for work or family on your hospital visit day to have a minimal impact on colleagues or family	−0.024	0.017	0.839	−0.023	−0.052	−0.050
You think about who is the best to ask if you need a favour	0.134	−0.047	0.812	−0.029	0.013	−0.022
You create priorities when you have many things to do	−0.084	0.130	0.679	−0.024	0.039	0.036
You make arrangements in your work and for your family to visit the hospital as planned	0.044	0.012	0.568	0.025	−0.036	0.184
You think about whether you impose excessive burdens on the person you ask for support	0.218	0.083	0.472	−0.042	0.115	−0.010
Factor IV: Ability to recognize and understand coping mechanisms						
You understand the specific ways to cope with any symptoms developed while in poor physical condition	0.026	−0.235	0.042	0.922	−0.006	0.101
You understand most causes of poor physical condition	−0.008	−0.094	−0.019	0.852	−0.077	0.096
You understand most of your physical conditions and patterns	0.021	0.003	−0.102	0.639	−0.056	0.200
You generally understand how to improve your physical condition	0.101	0.199	−0.072	0.624	0.098	−0.185
You have your original way to resolve symptoms	−0.026	0.137	0.033	0.605	0.104	−0.167
You control your daily life or physical condition mainly by using your original way	0.026	0.247	−0.019	0.486	0.069	−0.106
You control activities or restrict diet based on your original rule	−0.142	0.246	−0.044	0.391	0.061	0.108
Factor V: Ability to change objectives						
You had been worried about the future, but you changed your mind to appreciate every day	−0.165	−0.067	0.035	0.012	0.798	0.111
You had hesitated to act due to your worries, but you realized that it is no use worrying	0.008	−0.088	0.028	−0.031	0.744	0.037
You intentionally change your mind to do other things when you notice that you think deeply about cancer	−0.202	0.041	0.138	0.110	0.702	−0.022
You had tolerated your symptoms, but you think that you should tell someone about your difficulty	0.289	0.054	−0.108	−0.148	0.595	−0.003
You have begun to think that there are not only bad things that you have to tolerate, but also good things associated with cancer onset	0.106	−0.022	−0.047	0.100	0.589	−0.045
You had believed that you had to endure cancer treatment by yourself, but you have begun to think that you can take advantage of people's kindness	0.187	−0.037	−0.046	0.025	0.552	0.099
Factor VI: Ability to control physical function						
You pick the day when your condition is good to do what you need to do	0.091	0.010	−0.037	−0.005	−0.002	0.740
You ensure that you do not perform physically heavy tasks for a long time	−0.011	0.088	0.069	−0.037	0.021	0.719
You have a rest when your physical condition is poor, even if it is only for a short time	−0.015	−0.004	0.005	0.010	0.129	0.707
You keep the day free if you think you will be in poor physical condition	0.064	−0.014	0.198	0.060	−0.015	0.605
You secure time for rest as long as possible before and after important business	0.101	0.060	−0.085	0.137	0.007	0.588
Correlation between factors						
Factor II	0.579					
Factor III	0.340	0.541				
Factor IV	0.460	0.580	0.322			
Factor V	0.506	0.507	0.372	0.456		
Factor VI	0.546	0.559	0.444	0.391	0.378	

The mean adjustment ability score for the 47 items was 110.5 (*SD* 27.2; range 45–185) and the median score was 109.0. The correlation coefficient between the adjustment ability and subscale scores ranged from 0.65 to 0.84. The correlation coefficient between subscale scores ranged from 0.29 to 0.54 (Table 3).

**Table 3:** Correlation between adjustment ability score, subscale score and subscale correlation coefficient

Subscale	I	II	III	IV	V	VI
I Ability to tell someone						
II Ability to seek a better way to cope	0.548					
III Ability to increase certainty	0.375	0.565				
IV Ability to recognize and understand coping mechanisms	0.455	0.549	0.293			
V Ability to change objectives	0.494	0.456	0.346	0.435		
VI Ability to control physical function	0.562	0.594	0.456	0.446	0.415	
Adjustment ability score	0.798	0.844	0.653	0.689	0.668	0.760

### 4.4 Verification of reliability

Cronbach's α confidence coefficient across the adjustment ability scale was 0.952: Factor I α = 0.877; Factor II α = 0.909; Factor III α = 0.879; Factor IV α = 0.866; Factor V α = 0.842 and Factor VI α = 0.869.

The test–retest correlation coefficient between question items ranged from 0.442 to 0.696: Factor I ρ = 0.787; Factor II ρ = 0.742; Factor III ρ = 0.766; Factor IV ρ = 0.736; Factor V ρ = 0.776; Factor VI ρ = 0.725; and across 47 items ρ = 0.826. The intraclass correlation coefficient was .767.

### 4.5 Verification of validity (Table 4)

**Table 4:** Correlation coefficient between adjustment ability scale score, mental adjustment to cancer (MAC) scale score and functional assessment of cancer therapy‐general (FACT‐G) score

	Correlation coefficient							
	Subscale						Total adjustment ability score	
	I	II	III	IV	V	VI		
MAC								
Fighting spirit	0.360	0.323	0.245	0.270	0.476	0.256	0.426	0.477
Anxious preoccupation	0.138	0.382	0.236	0.181	0.135	0.208		
Fatalism	−0.135	−0.027	−0.093	−0.0091	−0.082	0.001		
Helplessness/hopelessness	−0.223	−0.057	−0.051	−0.156	−0.237	−0.114	−0.172	
Avoidance	−0.006	0.056	−0.024	−0.043	0.037	0.028	
Total score								
FACT‐G								
Physical	0.066	0.146	0.119	0.170	−0.014	0.206		
Social/familial	0.470	0.313	0.198	0.263	0.400	0.297		
Psychological	0.057	0.267	0.224	0.111	0.058	0.149		
Functional	0.257	0.153	0.087	0.164	0.277	0.094		0.152
Total score								

**Figure 1:**
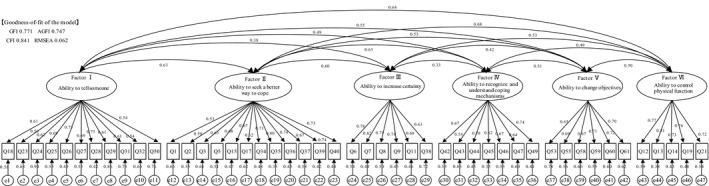
Results of confirmatory factor analysis. Decimal numbers indicate path coefficients. AGFI, adjusted goodness‐of‐fit index; CFI, comparative fit index; GFI, goodness‐of‐fit index; RMSEA, root mean square error of approximation

The correlation coefficient between the adjustment ability scale and the MAC scale was 0.477: fighting spirit score ρ = 0.426; and helplessness/hopelessness score ρ = –0.172. The correlation coefficient between the adjustment ability scale and MAC subscale was highest in “Ability to change objectives” and “Fighting spirit” (ρ = 0.476), followed by “Ability to seek a better way to cope” and “Anxious preoccupation” (ρ = 0.382). The correlation coefficient between the adjustment ability scale and FACT‐G was 0.152. The confirmatory factor analysis revealed the following: GFI = 0.771, AGFI = 0.747, CFI = 0.841 and RMSEA = 0.062 (Figure 1).

## 5 DISCUSSION

### 5.1 Investigation of reliability

Proper internal consistency of the scale was shown because Cronbach's α confidence coefficient was higher than 0.70 (reference value) across the adjustment ability scale and for each factor. Also, Spearman's rank correlation coefficient and intraclass correlation coefficient in test and retest items across the adjustment ability scale and for each factor was more than the reference value of 0.70 (range 0.73–0.83), indicating proper scale stability.

### 5.2 Investigation of validity

I believe that the scale's content‐related validity is ensured because I evaluated and revised the question items while preparing the proposed scale based on quantitative assessments, using a four‐point scale, by nurses who specialize in cancer care in their clinical practice as well as by university professors who are involved in cancer care. I also evaluated and revised the items based on responses from outpatients with cancer and concluded that the scale's face validity was established.

The correlation coefficient for the MAC scale (an external criterion) was.48. I believe that a constant discriminant validity is ensured because the correlation coefficient for the fighting spirit score—the MAC subscale that was expected to have a positive correlation with the adjustment ability scale score—was 0.426 and the correlation coefficient for the helplessness/hopelessness score—the subscale that was expected to have a negative correlation with the scale score—was −0.17. Conversely, the correlation coefficient for FACT‐G was 0.15, which indicates no association.

The goodness‐of‐fit for the model using confirmatory factor analysis ranged from 0.75 ‐ 0.84 for GFI, AGFI and CFI and was 0.062 for RMSEA, demonstrating poor fitness. Therefore, the construct validity could not be validated. Because it has been reported that the more observation variables there are, the smaller the GFI value is (Ishii, 2005), the number of items in this 47‐question scale could have had an impact on the GFI.

### 5.3 Characteristics of adjustment ability scale and practicality

One of the possible reasons why many female patients with breast cancer participated in this study was that most of the questionnaires were distributed to outpatients who were receiving anticancer drug treatment. The distribution location was considered to be associated with the sex of participants and the location of the cancer.

It is known that patients with cancer can find a new way to live, discover the meaning of life (Takeyama & Okamitsu, 2015) and seek out the purpose of life by creating a new life perspective after changing their old values and releasing their attachment to old feelings after cancer onset (Imaizumi, 2013). It has been demonstrated that cancer onset has a negative impact on patients with cancer, but factors including discovering a new perspective on life also can have a positive impact (Cheng, Sit, & Cheng, 2015). It is thought that an “Ability to change objectives” showed a higher correlation coefficient with a fighting spirit (a subscale of MAC) because of the ability of outpatients with cancer to change their values and way of living by addressing questions that require control. This ability is considered useful for these patients to live through their life after cancer onset. The highest correlation coefficient for anxious preoccupation (a subscale of MAC) was shown in “Ability to seek a better way to cope.” A previous study (Nakazawa et al., 2014) revealed that persistent physical symptoms may be associated with a patient's fear of having trouble in his/her basic social life as well as physical well‐being, a negative self‐image and a loss of meaning to his/her existence. It is thought that cancer patients ease the anxiety associated with recurrence and metastasis, characteristics of cancer and persistent physical symptoms by using their “Ability to seek a better way to cope,” because the anxiety is associated with a patient's basic social life and self‐image without being limited to physical concerns. In other words, using a patient's “Ability to seek a better way to cope” can be one of the ways he/she can properly cope with anxiety. Taken together, the adjustment ability scale is considered to be practical because it includes adjustment ability, which is a characteristic of outpatients with cancer and it can evaluate changes of the ability based on each subscale score.

## 6 STUDY LIMITATIONS AND FUTURE CHALLENGES

A possible limitation of this study is that the response period of retest after 2 weeks from the first response was not well managed because the questionnaire forms for the retest were sent with those for the first test and the construct validity was not validated enough.

This study revealed a high internal consistency, but Cronbach's α coefficient became higher as the number of question items increased. A reduced number of question items for ease of use and a steady internal consistency remain to be resolved. Moreover, I think it is necessary to clarify the association between the adjustment ability scale and factors that are thought to influence the adjustment ability. The relationship between use of adjustment ability, completion of treatment and continuous hospital visits in the future will also require further investigation.

## 7 CONCLUSION

I developed an adjustment ability scale consisting of six factors and 47 items. Cronbach's α coefficient indicated the scale's internal consistency and Spearman's rank correlation coefficient between test and retest revealed its stability. In preparation for the proposed adjustment ability scale, its content‐related validity and face validity were ensured. Its criterion‐related validity and discriminant validity were also confirmed by using external criteria, but its construct validity could not be validated.

## RELEVANCE TO CLINICAL PRACTICE

By using the adjustment ability scale developed in this research, we can express by numerical value the adjustment ability of outpatients with Cancer. By capturing the adjustment ability as a numerical value, it is possible to objectively extract a cancer patient who needs assistance for enhancing adjustment ability. Moreover, the effect of nursing support can be measured by the score of adjustment ability.

## CONFLICTS OF INTEREST

The authors have no funding or conflicts of interest to disclose.

## AUTHOR CONTRIBUTIONS

KH, SS: Study design. KH: Date collection and analysis. KH, SS: Manuscript preparation.
